# Recent exposure to ultrafine particles in school children alters miR-222 expression in the extracellular fraction of saliva

**DOI:** 10.1186/s12940-016-0162-8

**Published:** 2016-07-26

**Authors:** Annette Vriens, Tim S. Nawrot, Nelly D. Saenen, Eline B. Provost, Michal Kicinski, Wouter Lefebvre, Charlotte Vanpoucke, Jan Van Deun, Olivier De Wever, Karen Vrijens, Patrick De Boever, Michelle Plusquin

**Affiliations:** 1 Centre for Environmental Sciences, Hasselt University, Agoralaan, building D, 3590 Diepenbeek, Belgium; 2 Department of Public Health & Primary Care, Leuven University, Leuven, Belgium; 3 Environmental Risk and Health, Flemish Institute for Technological Research (VITO), Mol, Belgium; 4 Belgian Interregional Environment Agency, Brussel, Belgium; 5 Laboratory of Experimental Cancer Research, Department of Radiation Oncology and Experimental Cancer Research, Ghent University Hospital, Ghent, Belgium; 6 MRC/PHE Centre for Environment and Health, School of Public Health, Imperial College, London, UK

**Keywords:** Ultrafine air pollution, Children, Extracellular miRNA, Saliva

## Abstract

**Background:**

Ultrafine particles (<100 nm) are ubiquitous present in the air and may contribute to adverse cardiovascular effects. Exposure to air pollutants can alter miRNA expression, which can affect downstream signaling pathways. miRNAs are present both in the intracellular and extracellular environment. In adults, miR-222 and miR-146a were identified as associated with particulate matter exposure. However, there is little evidence of molecular effects of ambient air pollution in children. This study examined whether exposure to fine and ultrafine particulate matter (PM) is associated with changes in the extracellular content of miR-222 and miR-146a of children.

**Methods:**

Saliva was collected from 80 children at two different time points, circa 11 weeks apart and stabilized for RNA preservation. The extracellular fraction of saliva was obtained by means of differential centrifugation and ultracentrifugation. Expression levels of miR-222 and miR-146a were profiled by qPCR. We regressed the extracellular miRNA expression against recent exposure to ultrafine and fine particles measured at the school site using mixed models, while accounting for sex, age, BMI, passive smoking, maternal education, hours of television use, time of the day and day of the week.

**Results:**

Exposure to ultrafine particles (UFP) at the school site was positively associated with miR-222 expression in the extracellular fraction in saliva. For each IQR increase in particles in the class room (+8504 particles/cm^3^) or playground (+28776 particles/cm^3^), miR-222 was, respectively 23.5 % (95 % CI: 3.5 %–41.1 %; *p =* 0.021) or 29.9 % (95 % CI:10.6 %–49.1 %; *p =* 0.0027) higher. No associations were found between miR-146a and recent exposure to fine and ultrafine particles.

**Conclusions:**

Our results suggest a possible epigenetic mechanism via which cells respond rapidly to small particles, as exemplified by miR-222 changes in the extracellular fraction of saliva.

**Electronic supplementary material:**

The online version of this article (doi:10.1186/s12940-016-0162-8) contains supplementary material, which is available to authorized users.

## Background

Exposure to particulate matter (PM) is associated with adverse health outcomes such as an increased morbidity and mortality due to cardiovascular, respiratory and carcinogenic events [1–3]. The particles with an aerodynamic diameter smaller than 2.5 μm (PM_2.5_) are especially harmful since they can penetrate the body more deeply [4, 5]. It has been shown that inhaled ultrafine particles (UFP) with a diameter less than 0.1 μm can reach the circulation and are able to penetrate target organs other than the respiratory system [5–7], even crossing the blood–brain-barrier [8].

Micro-RNAs (miRNA) transfer signals to regulate gene expression at the posttranscriptional level and fine-tune the translation of mRNAs into proteins, both in health and disease [9]. They regulate cellular reactions in response to environmental insults and have been described as being responsive to PM exposure in humans [10–14]. The joining of the miRNAs with a carrier protects them from degradation in the extracellular environment. Additionally, miRNA mediated pathways operating via extracellular vehicles, such as microvesicles and protein complexes represent a potent cell-to-cell communication [15, 16] and can be influenced by PM exposure [11].

The present study investigates the association between children’s extracellular miRNAs in saliva and recent PM_2.5_ and UFP exposure. Saliva contains both buccal epithelial cells and leukocytes [17] as such it can be a non-invasive alternative, preferred to blood samples [18, 19]. Indeed, expression profiles of extracellular non-coding RNAs in saliva are similar to other fluids [20]. Furthermore, extracellular miRNAs are present in saliva [21] and saliva can be used to detect alterations at the molecular level in association with an external stressor [22]. We evaluate the expression of miR-222, which has a function in cell cycle and vascular biology as well as miR-146a which plays an important role in inflammation. Both have been shown responsive to PM exposure on a cellular level in adults [10, 12, 13]. To our knowledge, this is the first study investigating the effects of recent air pollution exposure on extracellular miRNA expression in saliva of children.

## Results

The study group of 80 participating children comprised 43 girls (53.8 %). The characteristics are given in Table 1. Briefly, age averaged 10.4 years (range 8.0–12.8) and BMI averaged 17.0 kg/m^2^ (range 12.7–23.4). Repeated measures were carried out for each individual. As such, the miRNAs were quantified in two samples that were collected at different time points.

**Table 1:** Characteristics of the study population (*n =* 80)

Variable		
Boys		37 (46.3 %)
Age, years		10.44 ± 0.97
BMI, kg/m^2^		17.01 ± 2.42
TV watching, hours/week		9.32 ± 5.46
Exposure to tobacco smoke		9 (11.2 %)
Maternal education		
	Low	26 (32.5 %)
	High	54 (67.5 %)

High maternal education was defined as college or university

Mean ± SD; frequency (%)

*SD* standard deviation, *BMI* body mass index

Table 2 gives an overview of the recent exposure parameters for fine and ultrafine particles, which were monitored at the school site, both indoor and outdoor. Daily average PM_2.5_ exposures at the school site were obtained by interpolation based on the school addresses. Indoor concentrations of UFP were on average 10300 particles/cm^3^ and PM_2.5_ averaged 4.6 μg/m^3^ in the examination room. At the playground, UFP was on average 32100 particles/cm^3^ and PM_2.5_ was on average 16.6 μg/m^3^. Daily PM_2.5_ at the day of the study visit averaged 24.2 μg/m^3^.

**Table 2:** Recent exposure to fine (PM_2.5_) and ultrafine (UFP) particles at the school site

Pollutant indicator	Mean (SD)	P25	P75	P90
*Indoor at school during the examination*				
UFP, #/cm^3^	10304 (6115)	5577	14081	18852
PM_2.5_, μg/m^3^	4.6 (3.6)	2.2	5.0	10.0
*Outdoor at school during the examination*				
UFP, #/cm^3^	32134 (21572)	15842	44618	67838
PM_2.5_, μg/m^3^	16.6 (16.7)	17.5	7.2	44.5
*Modeled daily residential PM*_*2.5*_				
Day of the examination (lag 0), μg/m^3^	22 (15.5)	11	26.3	42.5
Day before the examination (lag 1), μg/m^3^	19 (18.3)	7.4	24.2	40.1
Two days before the examination (lag 2), μg/m^3^	18.6 (20.6)	6.7	18.4	68.9
Average of 48h before the examination, μg/m^3^	18.8 (18.7)	7.9	20.8	57.8

*SD* standard deviation, *P25* 25^th^ percentile, *P75* 75^th^ percentile, *P90* 90^th^ percentile

We applied pollutant-specific mixed models to estimate the association of miR-222 and miR-146a expression levels in the extracellular fraction of saliva and exposure to UFP or PM_2.5_ (Table 3), while adjusting for the *a priori* selected covariates: sex, age, BMI, exposure to passive smoking, maternal education level, time and day of examination, time/week spent watching TV and the extracellular RNA concentrations. Because of the repeated measures design of the study, sampling and exposure measurements from two different time points were used to increase statistical power.

**Table 3:** The association between extracellular miRNA expression and recent exposure to fine (PM_2.5_) and ultrafine (UFP) particles

		miR-222			miR-146a		
Pollution indicator	IQR	Effect size	95 % CI	*p*-value	Effect size	95 % CI	*p*-value
*Indoor at school during the examination*							
UFP, #/cm^3^	8504	23.5	3.5–41.1	0.021	4.2	–9.4–17.8	0.54
PM_2.5_, μg/m^3^	2.8	4.7	−9.1–19.3	0.50	4.9	–4.6–14.8	0.31
*Outdoor at school during the examination*							
UFP, #/cm^3^	28776	29.9	10.6–49.1	0.0027	10.6	n/a	0.13
PM_2.5_, μg/m^3^	10.33	–2.3	11.5–7.1	0.63	–1.4	–7.4–4.6	0.65
*Modelled daily residential PM*_*2.5*_							
Day of the examination (lag 0), μg/m^3^	15.3	8.20	–5.8–22.4	0.26	–1.6	–11.6–8.5	0.76
Day before the examination (lag 1), μg/m^3^	16.8	6.92	–6.8–20.8	0.33	–4.9	–15–5.1	0.34
Two days before the examination (lag 2), μg/m^3^	11.7	3.96	–5.4–13.4	0.41	–2.4	–9.2–4.4	0.48
Average of 48h before the examination, μg/m^3^	12.9	5.31	–5.7–16.4	0.35	–3.5	–11.5–4.5	0.39

Estimated effects sizes are adjusted for school, time of the day, day of the week, age (continuous), gender, BMI (continuous), passive smoking, maternal education level, the hours TV/week and RNA content of the extracellular fraction. All estimates are represented as the % change in miRNA expression for an IQR increase in exposure to the pollutant

*P <* 0.05 was considered as a significant association

**Fig. 1:**
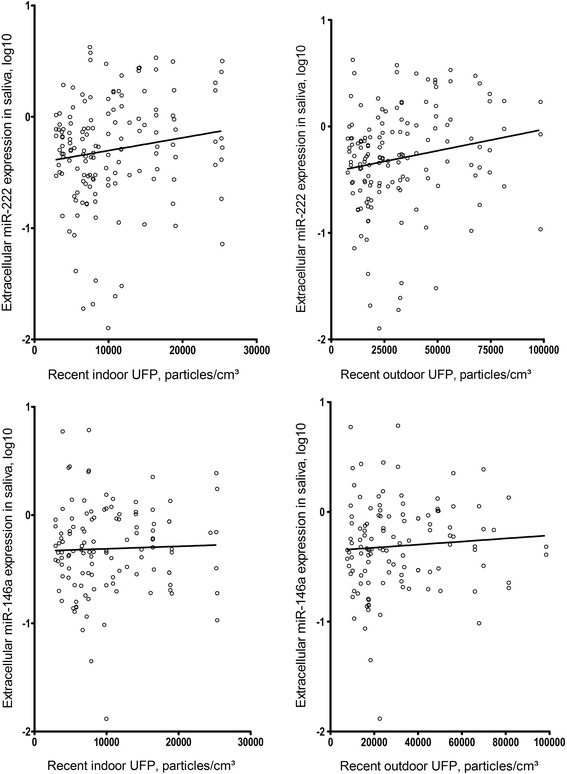
Correlation plot between recent UFP exposure and miR-222 and miR-146a. Plotting the miR-222 expression levels from the salivary extracellular fraction for the recent UFP exposure shows a positive association with both indoor (left) and outdoor (right) UFP

Recent UFP exposure was significantly associated with an increase in extracellular miR-222 expression in the saliva (Fig. 1). An IQR increment in indoor UFP concentration (+8504 particles/cm^3^) was associated with a 23.5 % increase (95 % confidence interval (CI): 3.5 %–41.1 %) in extracellular miR-222 levels (*p =* 0.021). Similarly, an IQR increment in outdoor UFP concentration (+ 28776 particles/cm^3^) was associated with a 29.9 % increase (95 % CI: 10.6 %–49.1 %) in miR-222 expression in saliva extracellular fraction (*p =* 0.0027). Daily average PM_2.5_ levels at the residence during the day of the study visit and the day before the study visit were not associated with extracellular miR-222. The mixed models did not show any association between air pollution exposure and miR-146a.

## Discussion

miRNAs are responsive to external stimuli, including PM [10–13] and contribute to cardiovascular disease development and progression [23–25]. Here, we focused on the extracellular levels of miR-222 as well as miR-146a, since both miRNAs have been shown responsive to PM exposure at the cellular level [10, 12, 13]. Furthermore, these miRNAs have been linked with the initiation and progression of atherosclerosis via endothelial dysfunction and inflammation [24, 26]. Air pollution is ubiquitous and ultrafine particles (<100 nm) translocate from the lung into the system and may contribute to adverse cardiovascular effects. In our repeated measure design, we found that miR-222 expression in saliva of children between 8–12 years of age was positively associated with exposure to ultrafine particles at school. Up-regulation of miR-222 is associated with vascular damage via decreased endothelial progenitor cell differentiation [27–30] and increased vascular smooth muscle cell cycling [31], as it targets c-Kit and its ligand stem cell factor [27] and p27kip1 [32]. miR-222 was reported to target eNOS in an indirect fashion [33] via direct inhibition of the transcription factor ets-1, which regulates eNOS expression [34, 35]. Additionally, since p27kip1 is an important regulator of cell cycle arrest, miR-222 has an oncogenic character [32]. This observation in the salivary extracellular fraction of children is consistent with observations in blood miRNA expression in steel workers [10]. An increased miR-222 expression in white blood cells (WBC) after three days of work was observed compared with baseline [10]. Additionally, controlled O_3_ exposure induced an increase in miR-222 expression in human sputum, which is a proxy for the respiratory system [36]. On the other hand, Motta and colleagues did not find an association between PM exposure and miR-222 expression in WBC in a subset of the steel workers population [12]. Furthermore, an inverse association between leukocyte miR-222 and 7-day exposure to PM_2.5_ and sulfate has been observed in the Normative Aging Study [13]. The latter two studies were performed in a population with different characteristics and exposure levels and in the case of Motta et al., a different technique (miRNA microarray) was used. Our results add to the evidence of a possible epigenetic mechanism via which cells are influenced by UFP or can respond to UFP, not only in heavily exposed adults, but also in children.

We did not find any significant changes in salivary extracellular miR-146a abundance in association with acute changes in UFP or PM_2.5_ exposure. Similarly, Bollati reported no association between PM and cellular miR-146a expression in three days post exposure samples of steelworkers compared with baseline, though miR-146a expression was inversely associated with PM metal compounds cadmium and lead [10]. However, Motta et al. performed miRNA microarray analysis on WBC in a subset of the steel plant population and found an up-regulation of miR-146a after three days exposure [12]. A study by Fossati found an inverse association between miR-146a expression in WBC and PM_2.5_ and black carbon exposure (7-days moving average) [13]. Possibly, miR-146a expression was not affected in our population or potential changes in miR-146a expression on a cellular level might not be reflected into the extracellular fraction of miRNAs. It should be clear from the above that the number of studies dealing with air pollution exposure and miRNA expression are limited and that maybe because of the different study designs, the results are hard to compare.

Extracellular miR-222 levels in saliva were significantly associated only with UFP, the smallest fraction of PM. However, using daily average PM_2.5_ exposure at the residence based on a land-use dispersion model showed a trend similar to the recent exposure to UFP analyses. A study by Nemmar and colleagues, showed that upon inhalation, UFP can already be detected in circulation after one minute and in extrapulmonary organs within 5 min [5]. Extracellular miR-222 is significantly associated with PM when considering the exposure shortly before the measurements.

MiRNAs in our study originate either from microvesicles or aggregated proteins which can be present in saliva. Differential ultracentrifugation as used in this study cannot separate vesicles from proteins [37]. However, to gain information about the content of the samples, we determined the size of the nanoparticles in a subset of the saliva samples by means of nanoparticle tracking analysis (Additional file 1). The particles with a size of 100 to 600 nm were most abundant (see Additional file 2), these can include exosomes, microvesicles or aggregated proteins [37]. In the future, studying specific fractions might further elucidate our results. The role of extracellular miRNAs and the microvesicles or protein complexes that bear them is not studied often in relation to air pollution. However, the limited evidence that is available, supports a role of extracellular mediators in the cardiovascular disease mechanisms after PM exposure. Using an experimental setup in mice, exposure to traffic-related PM led to an increased abundance of microvesicles with thrombogenic potential in plasma [38]. In a diabetic population, acute changes in PM_2.5_ were associated with a decrease of procoagulant microvesicles present in plasma [39]. Similarly, a study by Frampton and colleagues showed an increase in microparticles which expressed tissue factor after 2 and 5 days of PM_2.5_ exposure in diabetes type 2 patients [40].

Blood is a widely preferred biofluid for use in observational studies, nevertheless it can be challenging to collect blood samples in a population of children. Saliva on the other hand is minimally invasive, contains a diverse range of proteins, RNAs and miRNAs and may actually reflect an individual’s physiological condition [18, 19, 41]. Saliva may not only be used for diagnostic purposes, but it is also promising as indicator of local and systemic health.

The strengths of the present study are the repeated measures, which allow to control for intraindividual variation and a higher statistical power. Furthermore, recent exposure parameters were assessed at the school of the study participants and reflect at least to some extent a personal exposure since children spend much time at school. Our study is among the first to explore the effect of air pollution exposure on miRNA expression in children. Children are especially vulnerable because their body is not fully developed and cannot adequately cope with the toxic exposure to PM. Children tend to be more active and have a higher ventilation rate, therefore more particles can be deposited in their body [42, 43]. A limitation of this study is the explorative character of salivary extracellular miRNAs, as we choose to study miRNAs that were reported to be responsive in adult populations. Early studies on miRNA expression and PM exposure in humans highlighted the importance of miR-222 and miR-146a [10, 12–14]. These findings are supported by recent publications as well [44, 45]. However, studies using untargeted approaches indicated the involvement of wider range of miRNAs in the effects PM [46, 47]. Also, no targets of the miRNAs were profiled to indicate a downstream effect of altered miRNA expression.

## Conclusion

The present study provides evidence of an epigenetic response to UFP by alterations in the saliva extracellular miRNA abundance. Children had a higher expression of saliva extracellular miR-222 on days with higher ambient concentrations of ultrafine particulates (diameter smaller than 300 nm), compared to days with lower exposure. This rapid response was not observed with exposure parameters of larger particles, suggesting that UFP exposure is particularly relevant in the process of rapid adaptations of the extracellular miRNA content. However, the health consequences of altered extracellular expression levels miRNAs in response to air toxins remain to be elucidated.

## Methods

### Study population

This study was part of the COGNAC (COGNition and Air pollution in Children) study. Between 2011 and 2013, we invited children (grades three to six) from three primary schools in Flanders (Belgium) to participate in the study. The parents of participating children filled out a questionnaire including information about the current and previous residential addresses, the socioeconomic status of the family and the smoking behavior of the family members. In total the COGNAC study included 334 children recruited from three primary schools. For this specific study within the COGNAC children cohort, 80 children from two schools were randomly selected from the overall cohort. Among the 80 children, three sibling pairs and one twin pair was included. Each child collected saliva samples at two different time points, on average 11 weeks apart (first in November and second in January or the first week of February).

In order to rule out intra-individual diurnal variation, the three repetitive study visits were always scheduled on the same time during the day and the same day of the week. The examinations took place between November and February on Monday, Tuesday, Thursday, and Friday between 8:30 a.m. and 2:10 p.m.. All parents provided written informed consent for participation and oral assent of the children was renewed at each clinical examination. The COGNAC study was approved by the medical ethics committee of Hasselt University and the Eastern-Limburg Hospital, Belgium.

### Air quality assessment

#### Measurements of air pollutants at school

We used portable devices to measure ultrafine particles (UFPs) with a diameter 10–300 nm (Aerasense NanoTracer; Phillips, Eindhoven, The Netherlands), and particulate matter (PM with diameter < 2.5 μm) (AEROCET 531; MetOne Instruments Inc., Grants Pass, Oregon, US) in the school and at the playground on the examination days as part of the field work. The measurements were performed in the morning (9–12 a.m.). For each child, the measured outdoor pollution levels of the 10 min recess when children were at the school playground, before the study visit were used. Thus, outdoor exposure levels reflect ambient air pollution during the last time that the child was outside, which was approximately one hour before saliva donation. The measurements are significantly correlated with modeled concentrations at the school site (Additional file 1: Table S1).

#### Modeled PM_2.5_ concentrations at home address

We used a spatial temporal interpolation method to model the daily residential exposure levels (μg/m^3^) of PM_2.5_, at each child’s home address. This method takes into account land cover data obtained from satellite images (CORINE land cover data set) [48] and pollution data of fixed monitoring stations in combination with a dispersion model [49]. The model calculates the daily interpolated exposure concentrations in a high resolution receptor grid based on information from the Belgian telemetric air quality networks, point sources, and line sources. Overall model performance was evaluated by leave-one-out cross-validation. Validation statistics of the interpolation tool gave a spatial temporal explained variance of more than 0.80 for PM_2.5_ [50]. We used this model to estimate the residential exposure on the day (lag 1) of the examination, the day (lag 1) before the examination and two days (lag 2) before the examination as well as the average exposure of the 48 h before the examinations.

Since the parameters for fine and ultrafine PM were measured or interpolated in a exposure window ranging from hours to two days before sampling, they reflect recent exposure.

### Molecular measurements

#### Sample collection

Before sampling, subjects refrained at least 30 min from eating, drinking or hygienic procedures. Additionally, they rinsed three times with tap water to avoid contamination of the samples by food residues. Subjects had to collect 2 ml of whole saliva into the Oragene® RNA self-collecting kit (DNA Genotek Inc., Kanata, Ontario, Canada). The samples were immediately afterwards stabilized for RNA preservation. Within 6 h after sampling, samples were stored at -20 °C until further analysis.

### Isolation of extracellular miRNAs

Extracellular miRNA in saliva were isolated by differential centrifugation and ultracentrifugation of the samples. The protocol for isolation of the extracellular fraction was adapted from Théry et al [51], as such that it combined the sample processing, required for RNA stability when working with the DNA Genotek containers. After thawing, the Oragene® containers (DNA Genotek Inc., Kanata, Ontario, Canada) were incubated at 50 °C for one hour. Then, 1 ml aliquots were incubated at 90 °C for 15 min. To pellet the debris in the saliva, 40 μl of neutralizer solution (DNA Genotek Inc., Kanata, Ontario, Canada) was added to the samples and centrifuged at 1 500 x *g* for 10 min. The supernatant was collected and centrifuged at 16 000 x *g* for 20 min. Next, the supernatant was ultracentrifuged at 160 000 x *g* for one hour (Optima LE-80K Ultracentrifuge and ti70 fixed angle rotor; Beckman; Analis, Suarlée, Belgium). Polyallomer tubes for ultracentrifugation (Beckman; Analis, Suarlée, Belgium) were pre-treated with RNA*Zap* (Life Technologies, Gent, Belgium) to eliminate RNAse activity. Afterwards, the pellet was resuspended in 1x PBS (pH 7.4) and ultracentrifuged at 160 000 x *g* for one hour. The vesicle-containing pellets were resuspended in RNAse-free water and stored at -80 °C. The composition of the extracellular fraction and size distribution of the vesicles were evaluated using nanoparticle trafficking analysis in a subset of the samples (Nanosight Ltd.; Amesbury, UK) (Additional file 1).

miRNAs and larger RNA species were isolated using the miRNeasy mini kit (Qiagen; Valencia, California, USA) following the manufacturer’s instructions. After homogenization, the samples were spiked with 250 fmol *C. elegans miR-39* for normalization of the expression data [52, 53]. Total RNA and miRNA yield of the samples was quantified using Qubit assays (respectively Qubit br RNA assay and Qubit miRNA assay; Life Technologies; Ghent, Belgium). Furthermore, presence of miRNA was evaluated using small RNA Bioanalyzer (Agilent 2100; Agilent Technologies, Amstelveen, The Netherlands).

#### miRNA expression analysis

miR-222 and miR-146a were quantified using a two-step real-time PCR (RT-qPCR) with Taqman miRNA assays (Life Technologies). Reverse transcription was performed using 125 ng of total RNA input using looped primers (Megaplex RT primers human pool A & Taqman microRNA RT kit; Life Technologies) on a PCR gradient thermal cycler (TC-5000; Techne, Burlington, NJ, USA). cDNA synthesis ran 40 cycles of two minutes at 16 °C, one minute at 42 °C and one second at 50 °C; the reaction was inactivated at 85 °C for five minutes. cDNA samples were stored at -20 °C until qPCR analysis. Products of the reverse transcription were mixed with reagents of the Taqman miRNA assay and the Taqman Fast Advanced mastermix (Life Technologies) for quantification of the miRNAs. qPCR was carried out on ABI 7900HT sequence detection system (Applied Biosystems; Life Technologies) and thermal cycling was for 10 min at 95 °C, followed by 40 cycles of 15 s at 95 °C and one minute at 60 °C. Primer efficiencies of the Taqman assays were 108 % and 101 % for miR-222 and miR-146a, respectively. Efficiency of the *cel-miR-39* assay was 102 %. All runs were carried out in triplicate and with a no-template control (NTC) on 384-well plates with three inter run calibrators (IRC). Raw qPCR data were analyzed using the SDS Relative Quantification Software (version 2.3; Applied Biosystems). Cq values were transformed to a relative quantity against the external spike-in miRNA in qbase + software (Biogazelle; Zwijnaarde, Belgium).

#### Statistical analysis

Statistical analyses were carried out using SAS software (version 9.4; SAS Institute Inc., Cary, NC, USA). miRNA expression data were log_10_-transformed to obtain normal distribution of the data. The association between the air pollutants and extracellular miRNA expression data was assessed using a multivariate-adjusted mixed model taking in account possible confounders as well as the repeated measures by school and subject. Models were constructed based on *a priori* selected covariates including sex, age (continuous), BMI (continuous), exposure to passive tobacco smoke (categorical: yes/no), maternal education (categorical: low/high), time and day of examination, the hours/week spend watching TV (continuous), extracellular RNA concentration and school. High maternal education levels were defined as college or university. Time of the day was evaluated categorically (before 10 a.m., between 10 a.m. and 12 a.m. or after 12 a.m.) as well as day of the week. Q-Q plots of the residuals were used to test the assumptions of the models. All effect estimates were calculated as the percent change in extracellular miRNA expression associated with an IQR increment in the air pollutant concentration.

## Abbreviations

BMI, body mass index; cDNA, complement deoxyribonucleic acid; CI, confidence interval; COGNAC Study, cognition and air pollution in children study; eNOS, endothelial nitric oxide synthase; IQR, interquartile range; IRC, interrun calibrator; PCR, polymerase chain reaction; PM, particulate matter; UFP, ultrafine particles; WBC, white blood cells

## Additional files

Additional file 1: Characterization of extracellular fraction present in saliva of children. (DOC 31 kb) Additional file 2: Figure S1: Size distribution structures in the extracellular fraction of saliva. (JPG 85 kb)
