# Supplementary material for: Recent exposure to ultrafine particles in school children alters miR-222 expression in the extracellular fraction of saliva
Source: Environ Health. 2016 Jul 26;15:80. doi: 10.1186/s12940-016-0162-8 (PMC4962430; doi:10.1186/s12940-016-0162-8)
Supplement: Additional file 1: — Characterization of extracellular fraction present in saliva of children. (DOC 31 kb) [file 12940_2016_162_MOESM1_ESM.doc]

**Supplemental information**

**Characterization of extracellular fraction present in saliva of children**

***Nanoparticle Tracking analysis***

A subset of ten random samples was analyzed in order to determine the size distribution of the particles in the extracellular fraction. Nanoparticle Tracking Analysis (NTA) is carried out on extracellular particles diluted in PBS, using NanoSight LM10-HS (Nanosight Ltd., Amesbury, UK) equipped with a 405 nm laser and a CMOS camera system. Selected samples for NTA were analyzed in triplicate for 60 seconds. The temperature was monitored during the measurements. Data was analyzed with the NTA software (version 3.0), which generated three outputs with the size and concentration of the particles, which were averaged.

The presence of larger particles and the high quantity of particles in the samples resulted in high scattering, which causes great differences in the repeated NTA measurements. As such, large standard errors were observed between the measurements (Figure S1). NTA results also indicate an abundance of bigger particles in the samples, with predominance of particles of 200 nm and 500 nm in diameter (Figure S1). Due to the presence of large particles, the detection threshold was put at 4 and the camera level at 10. As such, the registration of the smallest particles (< 100 nm) can be withheld and as such they could not be observed. The NTA results indicate that the vast majority of the present vesicles is smaller than 600 nm (Figure S1).

We are aware that the fraction we yielded upon ultracentrifugation might contain a variety of structures that can harbor the miRNAs. Since the origin of the miRNAs was not further determined, we prefer to refer to it as extracellular miRNAs from saliva.

Figure S1: **Size distribution structures in the extracellular fraction of saliva.** The size distribution of the particles present in the extracellular fraction of saliva was estimated by Nanoparticle Tracking Analysis (NTA). The frequency for each particle size was obtained by averaging the concentrations from eight random samples. The concentrations were plotted against the particle size. Variation among the samples is represented by the red bars, reporting the standard error of the mean.

**Table S1**: Correlation between on-site measurements and interpolation data at the school.

| **On-site measurement the study** | **Daily mean on the day of the study** | **r** | **p-value** |
| --- | --- | --- | --- |
| UFP | PM_2.5_ | 0.52 | <0.001 |
| PM_2.5_ | PM_2.5_ | 0.64 | <0.001 |

UFP= ultrafine particles, PM_2.5_ = PM with an aerodynamic diameter less than 2.5 µm, r=Spearman correlation coefficient
