# Supplementary figures and images for: Recent exposure to ultrafine particles in school children alters miR-222 expression in the extracellular fraction of saliva
Source: Environ Health. 2016 Jul 26;15:80. doi: 10.1186/s12940-016-0162-8 (PMC4962430; doi:10.1186/s12940-016-0162-8)

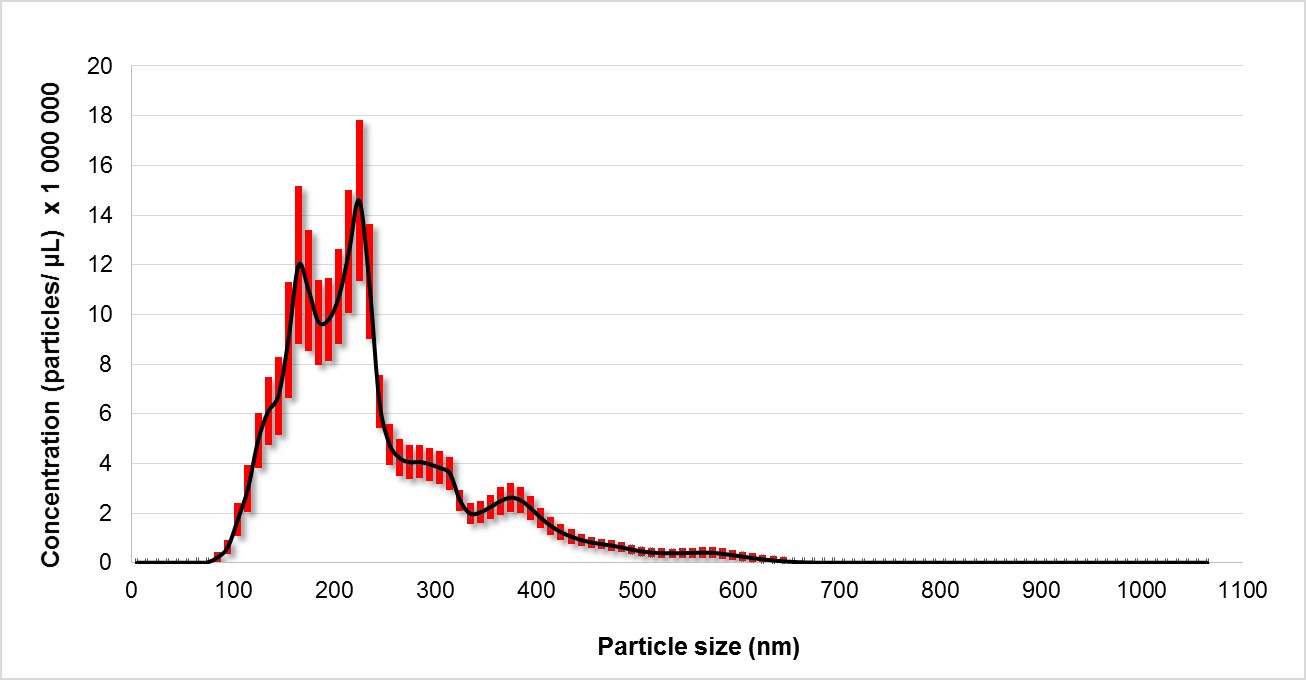

Supplement: Additional file 2: Figure S1: — Size distribution structures in the extracellular fraction of saliva. (JPG 85 kb) [file 12940_2016_162_MOESM2_ESM.jpg]
